# The Complex Construct of Wellbeing and the Role of Vagal Function

**DOI:** 10.3389/fnint.2022.925664

**Published:** 2022-07-07

**Authors:** Lowri Wilkie, Zoe Fisher, Andrew H. Kemp

**Affiliations:** ^1^School of Psychology, Faculty of Medicine, Health and Life Science, Swansea University, Swansea, United Kingdom; ^2^Regional Neuropsychology and Community Brain Injury Service, Morriston Hospital, Swansea, United Kingdom; ^3^Health and Wellbeing Academy, Faculty of Medicine, Health and Life Science, Swansea University, Swansea, United Kingdom

**Keywords:** heart rate variability, vagal function, emotion, self-connection, social-connection, nature-connection, climate psychology

## Introduction

The construct of wellbeing has been difficult to define and has avoided straightforward solutions (Bache et al., 2016). This also makes the topic a fascinating one to reflect on and study. Here we introduce the complex construct of wellbeing, then discuss how the vagus nerve might support the experience of it, before reflecting on some complexities and opportunities arising from a focus on vagal function as target for “inner development” and transformation of the self.

## What is Wellbeing?

While illbeing and wellbeing are partly related, they are also distinct (Westerhof, 2010; Fisher et al., 2020; Hunter, 2020). This claim is supported by findings that people living with chronic conditions including mental illness (Goodman et al., 2018), heart disease (Magán et al., 2021) or acquired brain injury (Wilkie et al., 2021) have tremendous capacity to improve the determinants of wellbeing (see also Kemp et al., 2022). The field of positive psychology initiated its study on “human flourishing” using a concept of wellbeing defined purely by positive experiences. While these early foundations oriented psychology away from a skewed focus on dysfunction and illbeing, later “waves” of positive psychology and the more heterogeneous discipline of wellbeing science are expanding the focus to existential positive psychology (Wong, 2019; Wong et al., 2021), collective (social) (Atkinson et al., 2020; Waters et al., 2021) and planetary wellbeing (Antó et al., 2021; Community et al., 2021) (Lomas et al., 2020; see also Lomas, 2022). These developments reveal the need to redefine “wellbeing” in a more holistic and transdisciplinary manner.

## Wellbeing as “Connection” SUPPORTED by Healthy Vagal Function

We have captured a role for the vagus in wellbeing in the development of our “GENIAL” model (**G**enomics–**E**nvironment–vagus **N**erve–social **I**nteraction–**A**llostatic regulation–**L**ongevity), an evidence-based framework that integrates multi- and interdisciplinary findings from wellbeing science (Kemp et al., 2017; Fisher et al., 2020; Mead et al., 2021; Kemp and Fisher, 2022). The vagus nerve is the tenth cranial nerve that connects many different organs including heart, gut, liver and lungs and the primary nerve supporting the parasympathetic nervous system. This nerve is an ideal candidate for supporting wellbeing, given its regulatory role over the sympathetic nervous system (Porges, 2011; Deuchars et al., 2018), hypothalamic-pituitary adrenal axis (Porges, 2011; Keller et al., 2021), immune system (Tracey, 2002; Pavlov and Tracey, 2012) and gut-brain axis (Forsythe et al., 2014; Fülling et al., 2019). Critically, heart rate variability (or HRV) reflects an index of the functioning of the vagus nerve (Paso et al., 2013), and can be used as a target for tracking the impact of wellbeing-related interventions including for example, a healthy lifestyle (Jandackova et al., 2019), HRV biofeedback (Lehrer et al., 2020), mind-body techniques (Zou et al., 2018), compassion focused therapy (Bello et al., 2020), loving kindness meditation (Kok et al., 2013) and nature-based wellbeing interventions (Richardson et al., 2016).

We have argued that “wellbeing” needs to take into account the self, other people and the planet (Fisher et al., 2020; Kemp and Fisher, 2022; Kemp et al., 2022). Furthermore, if we were pressed to define “wellbeing” in a single word, we would suggest the word “connection”, which encapsulates (1) self-connection, (2) social-connection and (3) nature-connection. We further suggest that “connection” may be supported and promoted by functioning of the vagus. This suggestion is supported by Porges” polyvagal theory (Porges, 2011, 2021), which characterizes the (myelinated) vagus as the foundation for the social engagement system, facilitating the experience of safety outside of awareness (neuroception). Higher levels of vagally-mediated HRV will therefore facilitate the experience of safety and increase capacity for connection and wellbeing, and we note above that HRV may provide a specific target for improving wellbeing-related outcomes. We now review key concepts relating to self-connection, social-connection and nature-connection, highlighting a role for vagal function in each.

## Connection to Self and the Promotion of Individual Wellbeing

“Self-connection” is rooted in the awareness of oneself, and the acceptance and alignment of behavior based on that awareness (e.g., Klussman, 2021). Acceptance and Commitment Therapy (ACT), for example, utilizes mindfulness, acceptance, awareness and value-based action (i.e., behavioral alignment) to bring about a more connected (transcendent) self (Edwards et al., 2019; Hayes, 2019). We further suggest that self-connected individuals will display stronger mind-body integration, indexed through higher levels of vagal function. Eighty percent of vagal nerve fibers are afferent in nature, which carry information from the viscera to the brain (Goehler et al., 2000; Saper, 2002) and provide a mechanism by which health behaviors can impact on psychological experience. One example is how the vagus uses interoception to differentiate between healthy and pathogenic bacteria in the gut, then relays this information to the brain, modulating stress and inflammation (Bonaz et al., 2018; Breit et al., 2018; Fülling et al., 2019). Vagal function is also associated with various other relevant constructs to self-connection including positive emotion (Kok et al., 2013), meaning in life (Dang et al., 2021), compassion (Bello et al., 2020), emotional regulation (Mather and Thayer, 2018), self-rated health (Jarczok et al., 2015) and psychological flexibility (Kashdan and Rottenberg, 2010; Pinna and Edwards, 2020).

## Connection to Others and Promotion of Collective Wellbeing

Self-connection may also facilitate collective wellbeing, which is defined as a group of individuals who independently and interdependently feel good and function well (Allison et al., 2021). For example, social relational emotions including compassion, gratitude and awe support an individual's capacity to connect with others and are powerful determinants of prosocial behavior (Bartlett and DeSteno, 2005; Grant and Gino, 2010; Lindsay and Creswell, 2014; Stellar et al., 2017). Moreover, vagal function has been shown to increase perceived connectedness, subsequently increasing positive emotions, and further increasing vagal function in an upward spiral relationship (Geisler et al., 2013; Kok et al., 2013). According to polyvagal theory (Porges, 2011, 2021), the myelinated vagus in combination with other cranial nerves provides a “vagal brake” over the phylogenetically older sympathetic nervous system and unmyelinated vagus facilitating social engagement and interaction, and this process is outside of awareness. Acute augmentation of oxytocin—a neuropeptide that plays a regulatory role in human social behavior—has been shown to increase HRV at rest without a change in mood (Kemp et al., 2012), a finding interpreted as facilitating the capacity for social engagement. Further insights on the role of HRV in social-connection are revealed in research on cardiac synchrony in romantic couples (Coutinho et al., 2021). Negative (antiphase) synchrony of HRV was reported regardless of whether discussion was positive or negative, and these findings were interpreted to reflect a “physiological dance of co-regulation”, in which partner A decreased their ability to self-regulate arousal (decreased HRV) while partner B increased their own ability (increased HRV). Social-connection has been described as a basic psychological need that is critical for individual wellbeing (Baumeister and Leary, 1995; Deci and Ryan, 2014) and may also lay the foundations for connecting to non-human others (i.e. nature) (Petersen et al., 2019), a topic we turn to next.

## Connection to Nature and Promotion of Planetary Wellbeing

Social relational emotions (such as awe) may directly contribute to the experience of wellbeing when in nature (Anderson et al., 2018; Petersen et al., 2019). As with social connectedness, nature-connection may also reflect a basic psychological need (Baxter and Pelletier, 2019). A recently published pragmatic-controlled trial of “forest bathing” (McEwan et al., 2021) reported improvements in positive emotions, nature connection and compassion, but interestingly, only 57% of participants displayed an increase in HRV. The authors attributed HRV decreases to the experience of biophobia, consistent with the evolutionary model of wellbeing (Richardson et al., 2016), which highlights how the natural environment may also trigger “primed” emotional responses associated with anxiety in some people. Despite this variability across participants, findings from meta-analysis (Richardson et al., 2016) have shown that natural environments promote greater vagal function relative to urban environments. Feelings of belonging, togetherness and connection not only support capacity for nature-connection but may also provide a basis for caring about climate-related matters (Stoknes, 2015; Pihkala, 2022), providing systems-informed opportunities for connecting wellbeing with major societal challenges such as the climate emergency.

## The Relationship Between Vagal Function and Wellbeing

We suggest here that vagal function may provide an upstream mediator of pathways to wellbeing, rather than a direct proxy of it. While there is a significant body of evidence linking vagal function to aspects of wellbeing, there is less support for a *direct link*. Several large studies have now reported no direct associations with wellbeing (Sloan et al., 2017; Tegegne et al., 2018; Behnke et al., 2022). There are several potential explanations for these contradictory findings including definitional issues and methodological considerations.

Wellbeing is a complex construct and it is possible therefore that vagal function is associated with some aspects of wellbeing (e.g., physical health, social connection) more than others. For example, a study on over 8 million fitbit users (Natarajan et al., 2020) reported that young people who took 12,000–13,000 steps a day displayed 21.2% (males) and 22.9% (females) greater high frequency HRV than those who took 5,000–6,000 steps. However, it is important not to overlook the bidirectional relationships between mental and physical health, and the key role of HRV in this regard (Kemp and Quintana, 2013). There are also methodological considerations when conducting HRV research, including adequate control of potential confounding, especially when certain variables cannot be randomly allocated to groups (Miller and Chapman, 2001). This situation can lead to statistical anomalies including Simpon's paradox, which refers to the diminution or enhancement of an association between two variables (e.g. HRV and wellbeing) when a third confounding variable is “corrected” or “controlled” (e.g., age, sex, psychopathology) (Miller and Chapman, 2001; Tu et al., 2008). Novel analytical methods such as propensity score weighting using generalized boosted modeling may help to overcome the limitations of traditional analysis (e.g., Kemp et al., 2014), but are yet to be applied to associations between HRV and wellbeing. Finally, there is a need to move beyond simplistic models and toward more sophisticated models that better reflect the complexity and inter-relationships between various proposed mechanisms and their moderators, highlighting a need for future work involving causal path modeling to further explore putative relationships. A related point is the potential for low coherence between physiological responses and subjective report, which may reflect denial or poor interoceptive capacities (Vella-Brodrick et al., 2022). In summary, we suggest that HRV may provide an upstream mediator of wellbeing, rather than a surrogate measure of it.

## Discussion and Conclusions

The science of wellbeing has given rise to new opportunities including interventions designed to improve wellbeing in people living with chronic conditions (e.g., Tulip et al., 2020; Wilkie et al., 2021; Gibbs et al., 2022), for whom interventions have typically focused on reducing illbeing. Wellbeing in chronic conditions may be promoted through efforts to tackle wellbeing at multiple levels of scale for system-wide change, consistent with a systems-informed positive psychology (Kern et al., 2020). For instance, we found that a group-based surf therapy intervention for people with acquired brain injury involving a partnership across academia, healthcare and community-based organizations may facilitate a cascade of mechanisms supporting positive change (Wilkie et al., 2021; Gibbs et al., 2022). This provides a concrete example of how our GENIAL model has been applied, taking into consideration individual, community, environment and behavior change while tackling entrenched socio-structural barriers (e.g., financial constraints, disciplinary silos) (see also Kemp and Fisher, 2022). As well as focus on the individual and groups, our partnerships enable us to bridge the gap between statutory services and local community resources capable of facilitating the determinants of wellbeing at a higher level of scale.

As the impacts of modern living on the natural environment continue to worsen (Ripple et al., 2021), wellbeing interventions that align with “sustainable wellbeing”—the promotion of wellbeing that does not come at a cost to other people or the natural environment (O'Brien, 2016)—are needed. Research shows that spending time in the natural environment—an activity that that has been shown to improve vagal function—supports individual as well as collective wellbeing (Waters et al., 2021) while also promoting pro-environmental behaviors (Martin et al., 2020). The therapist of the future will need to embrace emerging frameworks such as the power threat meaning framework (Johnstone and Boyle, 2018), that support non-anthologizing understandings of different reactions to societal challenges including climate breakdown (Morgan et al., 2022), which is expected to have devastating impacts on health and wellbeing (Fritze et al., 2008; Ellis and Albrecht, 2017; Kelman et al., 2021). Vagal function may provide an appropriate target for objectively measuring the impacts of a wide range of interventions to support connections to self, others and nature, laying the foundations for transformation of the self and progress on the “inner development goals” which are now seen as essential for making progress on the “sustainable development goals” (Wamsler and Brink, 2018; Woiwode et al., 2021) [see also: https://www.innerdevelopmentgoals.org/].

## Author Contributions

All authors listed have made a substantial, direct, and intellectual contribution to the work and approved it for publication.

## Conflict of Interest

The authors declare that the research was conducted in the absence of any commercial or financial relationships that could be construed as a potential conflict of interest.

## Publisher's Note

All claims expressed in this article are solely those of the authors and do not necessarily represent those of their affiliated organizations, or those of the publisher, the editors and the reviewers. Any product that may be evaluated in this article, or claim that may be made by its manufacturer, is not guaranteed or endorsed by the publisher.
